# NanoFe_3_O_4_ as Solid Electron Shuttles to Accelerate Acetotrophic Methanogenesis by *Methanosarcina barkeri*

**DOI:** 10.3389/fmicb.2019.00388

**Published:** 2019-03-05

**Authors:** Li Fu, Ting Zhou, Jingyuan Wang, Lexing You, Yahai Lu, Linpeng Yu, Shungui Zhou

**Affiliations:** ^1^Fujian Provincial Key Laboratory of Soil Environmental Health and Regulation, College of Resources and Environment, Fujian Agriculture and Forestry University, Fuzhou, China; ^2^College of Urban and Environmental Sciences, Peking University, Beijing, China

**Keywords:** magnetite nanoparticle, acetotrophic methanogenesis, *Methanosarcina barkeri*, electron shuttles, wetland

## Abstract

Magnetite nanoparticles (nanoFe_3_O_4_) have been reported to facilitate direct interspecies electron transfer (DIET) between syntrophic bacteria and methanogens thereby improving syntrophic methanogenesis. However, whether or how nanoFe_3_O_4_ affects acetotrophic methanogenesis remain unknown. Herein, we demonstrate the unique role of nanoFe_3_O_4_ in accelerating methane production from direct acetotrophic methanogenesis in *Methanosarcina*-enriched cultures, which was further confirmed by pure cultures of *Methanosarcina barkeri.* Compared with other nanomaterials of higher electrical conductivity such as carbon nanotubes and graphite, nanoFe_3_O_4_ with mixed valence Fe(II) and Fe(III) had the most significant stimulatory effect on methane production, suggesting its redox activity rather than electrical conductivity led to enhanced methanogenesis by *M. barkeri*. Cell morphology and spectroscopy analysis revealed that nanoFe_3_O_4_ penetrated into the cell membrane and cytoplasm of *M. barkeri*. These results provide the unprecedented possibility that nanoFe_3_O_4_ in the cell membrane of methanogens serve as electron shuttles to facilitate intracellular electron transfer and thus enhance methane production. This work has important implications not only for understanding the mechanisms of mineral-methanogen interaction but also for optimizing engineered methanogenic processes.

## Introduction

Acetate is a major intermediate product in the anaerobic digestion process and can be further metabolized by direct or syntrophic pathways to form CH_4_, the end product of anaerobic digestion. In the syntrophic pathway, acetate is oxidized to H_2_ and CO_2_ by syntrophic acetate-oxidizing bacteria, and H_2_ is consumed by hydrogenotrophic methanogens to generate CH_4_ (Schnürer et al., 1999). The syntrophic pathway often dominates in thermophilic anaerobic digesters (Hao et al., 2010). In the direct pathway, acetotrophic methanogens like *Methanosarcina* and *Methanosaeta* convert acetate into CH_4_ and CO_2_ (Welte and Deppenmeier, 2014), which is the main acetate degradation pathway in mesophilic anaerobic digesters.

Magnetite is a common low-toxicity ferromagnetic black color iron oxide that is widely distributed on earth. Magnetite has a relatively low redox potential (-314 mV) and both Fe(II) and Fe(III) are present in its structure. There are 16 octahedral voids in the magnetite unit cell, with 8 Fe^2+^ and 8 Fe^3+^ filled in the octahedral void (Fleet, 1981). Magnetite has an excellent conductivity because Fe^2+^ and Fe^3+^ are basically disorderly arranged on the octahedron and electrons can rapidly transfer between the two oxidation states of iron. Magnetic nanoparticles (nanoFe_3_O_4_) have been widely used in the catalytic (Hudson et al., 2014), biomedical (Mohammed et al., 2017), and environmental fields (Mohammed et al., 2017; Su, 2017).

Magnetite has been demonstrated to stimulate methane production in enrichment cultures initiated with rice paddy soil (Lovley, 2017). The authors hypothesized that magnetite mediated direct interspecies electron transport between the *Geobacter* species and the *Methanosarcina* species to promote methane production (Kato et al., 2012). Subsequently defined coculture studies with *G. metallireducens* and *M. barkeri* validated the hypothesis that magnetite promotes DIET (Rotaru et al., 2014; Tang et al., 2016). Until now, many studies have suggested that the addition of either magnetite or other conductive materials such as graphite, activated carbon, etc., to promote DIET can significantly facilitate syntrophic methanogenesis from benzoate (Zhuang et al., 2015), butyrate (Li et al., 2015; Zhang and Lu, 2016; Fu et al., 2018), propionate (Viggi et al., 2014; Yamada et al., 2015; Jing et al., 2017), acetate (Yamada et al., 2015; Zhuang et al., 2018), or ethanol (Kato et al., 2012; Rotaru et al., 2014). How magnetite mediated DIET has been a major concern for researchers. Current evidence suggests that magnetite can partially substitute and supplement the function of the OmcS cytochrome on the pili of *G. sulfurreducens*, but it cannot substitute the pili itself (Lovley, 2017). It is undoubtedly an important discovery that magnetite exhibits the function of cytochromes that are involved in DIET. This indicates that magnetite can act as a solid-state electron shuttle to mediate DIET in syntrophic methanogenesis.

The accelerated syntrophic acetate oxidation with magnetite has been confirmed under conditions of a high ammonium concentration (Zhuang et al., 2018) or high temperature (Yamada et al., 2015). Although magnetite also accelerated the mesophilic methanogenesis from acetate (Yang et al., 2015), there are still some problems that remain not resolved. First, does magnetite make the syntrophic acetate oxidation as the dominant methanogenesis pathway? Second, can magnetite promote the direct acetotrophic methanogenesis? To address these problems, an enriched culture system was incubated with ^13^C isotope-labeled acetate as the sole substrate to trace the active microorganisms and clarify the methanogenesis pathway. We found that magnetite played a unique role in promoting the acetotrophic methanogenesis by *Methanosarcina barkeri*, whereas carbon nanotubes (CNTs) or graphite nanoparticles with a higher electrical conductivity did not have the same capability. Pure culture experiments of *M. barkeri* were carried out to further explore the underlying mechanism of magnetite.

## Materials and Methods

### Enrichment Cultivation

The surface soil samples were collected on 25 July 2012 from an open fen close to the Wetland National Nature Reserve of Zoige located in Qinghai-Tibetan Plateau (33°47^′^ N, 102°57^′^ E) (Fu et al., 2015). Enrichment cultivation was conducted in the same way as previously described (Fu et al., 2018). Enrichment incubation was initiated by inoculating 4% (v/v) pre-incubated soil slurry (21 days) into 60-mL vessels containing 25 mL of Hepes-buffered (30 mM, pH 7.0) fresh medium under a headspace of N_2_/CO_2_ (80/20). The basal medium contained MgCl_2_⋅6H_2_O (0.4 g L^-1^), CaCl_2_⋅H_2_O (0.1 g L^-1^), NH_4_Cl (0.1 g L^-1^), KH_2_PO_4_ (0.2 g L^-1^), KCl (0.5 g L^-1^), and resazurin (0.0005 g L^-1^), and was supplemented with Na_2_S⋅9H_2_O (1.0 mM), vitamin and trace element solutions as described previously (Lü and Lu, 2012). Sodium acetate was added to a final concentration of 5 mM in the initial four transfers and then increased to 10 mM thereafter. Cysteine was not added to avoid the possible effect of electron shuttle molecules. NanoFe_3_O_4_ were synthesized as described previously (Kang et al., 1996). The first transfer was inoculated from the pre-incubated soil slurry, the effect of nanoFe_3_O_4_ concentration (2.32, 4.64, and 6.96 mM of Fe in the medium) was determined. Continuous transfers were conducted in the presence of nanoFe_3_O_4_ (4.64 mM of Fe in the medium). The inocula for every transfer were taken from the later nanoFe_3_O_4_-amended cultivation. For a comparison, the same inocula were used to make parallel preparations without nanoFe_3_O_4_ in the medium (i.e., the control).

### Isotope Labeling and Molecular Analysis

The final cultivation (after thirteen transfers) was used to extract microbial DNA following the previous protocol (Ma et al., 2012). Briefly, 10 mL of enrichment was extracted sequentially with TPMS buffer [50 mM Tris–HCl (pH 7.0), 1.7% (wt/vol) polyvinylpyrrolidone K25, 20 mM MgCl_2_, 1% (wt/vol) sodium dodecyl sulfate] and phenol-based lysis (PBL) buffer [5 mM Tris–HCl (pH 7.0), 5 mM Na_2_EDTA, 1% (wt/vol) sodium dodecyl sulfate, 6% (vol/vol) water-saturated phenol]. Beads-beating was performed in FastPrep-24 (MP Biomedicals, United States). The supernatants were further extracted with water-saturated phenol, phenol-chloroform-isoamyl alcohol [25:24:1 (vol/vol/vol)], and chloroform-isoamyl alcohol [24:1 (vol/vol)]. The extracts were purified by cold ethanol and sodium acetate.

DNA samples from both the control and nanoFe_3_O_4_ treatment were used to construct bacterial and archaeal clone libraries. The PCR amplification, cloning and sequencing followed the previous procedure (Kumar et al., 2016). Phylogenetic trees were constructed using the neighbor-joining algorithm of the MEGA7 program (Kumar et al., 2016), and bootstrap analysis was implemented with 1000 replicates. DNA-SIP was performed using the same cultivation. For this purpose, the sodium acetate-2-^13^C (99 atom%; Sigma-Aldrich) was added as the substrate. At the end of incubation, the carbon isotopic ratios (δ^13^C values) of CH_4_ and CO_2_ were analyzed by a gas chromatography-isotope ratio mass spectrometry system (Fu et al., 2018). DNA was extracted from the ^13^C-labeled and non-labeled cultivations and subjected to DNA-SIP procedure through the isopycnic centrifugation and density gradient fractionation of DNA as described previously (Rui et al., 2011). Centrifugation medium was prepared by mixing cesium trifluoroacetate (CsTFA) (Amersham Pharmacia Biotech) with gradient buffer (0.1 M Tris–HCl, pH 8; 0.1 M KCl; 1 mM EDTA). The mixtures were centrifuged in a Ti90 vertical rotor (Beckman) at 177000 *g*, 20°C for >36 h using Beckman Optima 2-80XP Ultracentrifuge (Beckman Coulter, United States). The density-resolved DNA was fractionated, and the buoyant density of each fraction was determined by refractometer.

The density-resolved DNA gradients were quantified for total bacteria and archaea using real-time quantitative PCR (Gan et al., 2012). Quantitative PCR of archaeal and bacterial 16S rRNA genes were carried out in a 7500 real-time PCR system (Applied Biosystems) using the primer pair Ar364/Ar934, and Ba519f/Ba907r, respectively. The fingerprinting of the DNA gradients was conducted using the terminal restriction fragment length polymorphism analysis (T-RFLP) following the protocol described previously (Liu et al., 2011). PCR amplification was performed using the primer pairs of Ba27f/Ba907r for bacteria and Ar109f/Ar934r for archaea. The 5^′^ end of the Ba27f and Ar934r primers were labeled with 6-carboxyfluorescein (FAM). PCR products were purified using an agarose gel DNA extraction kit (TaKaRa) and digested with *Msp* I (Takara) for bacteria and *Taq* I (Takara) for archaea, respectively.

### Pure Culture Experiments

*Methanosarcina barkeri* (DSM800) were purchased from German culture collection DSMZ (Braunschweig, Germany). The basal medium was consistent with that described in the previous enrichment culture experiment. Sodium acetate was added to a final concentration of 10 mM. The effect of nanoFe_3_O_4_ was tested for pure culture strains.

### Electrochemical Analysis

The redox activity was analyzed by cyclic voltammetry (CV) and electrochemical impedance spectroscopy (EIS) using the CHI660 as described previously (Zhou et al., 2018). Graphite plates (1.0 cm × 1.5 cm) and saturated calomel reference electrodes (SCE) were used as the working electrode, counter electrode and reference electrode, respectively. The CV scan parameters were as follows: initial potential was 0.6 V vs. SCE, maximum potential was 0.6 V vs. SCE, minimum potential was -0.8 V vs. SCE, termination potential was 0.6 V vs. SCE, and the scan rate was 0.015 V/s. The EIS scan had a high frequency value of 100000 Hz, a low frequency value of 0.1 Hz, and an amplitude of 0.005 V. In the electrochemical impedance test, the protoplast part of the biofilm was considered to be insulated under low frequency electrical perturbations, and the biofilm was considered as a multi-element circuit.

### XRD and Raman Analysis

For XRD analysis, the culture was centrifuged (4000 *g*, 2 min). Then the supernatant was removed, and the solids were lyophilized in vacuum for 48 h. The dried sample was analyzed by a D/MAX-2400 X-ray diffractometer. The monochromatic Cu target Kα was irradiated with a current of 80 mA and a voltage of 40 kV. The acquisition range is 3–70° with an accuracy of 1.2°/min and a time of 6 s (Li et al., 2015). For Raman analysis, the centrifuged sample was air-dried under nitrogen protection until no significant aqueous layer was visible. Mineral structure and composition were analyzed using a LabRam HR800 laser confocal micro-Raman spectrometer. The excitation wavelength is 532 nm, the grating is 600 gr/mm, the objective magnification is 50 times, and the focused spot size is about 1.5 μm. The entire process is carried out under nitrogen protection.

### Microscopy

For scanning electron microscope (SEM) observation, cells were carefully harvested without stir. Following centrifugation (4000 *g*, 2 min), the supernatant was removed and the pellet was fixed with 2.5% (wt/vol) glutaraldehyde, dehydrated using a graded series of ethanol solutions, and dried with t-butanol. The samples were mounted on copper stubs, coated with platinum, and then imagined using JEOL S-4800 (JEOL, Japan). For transmission electron microscope (TEM) analysis, the fixed cells were sent to Beijing Zhongkebaice Technology Service Co., Ltd. to make ultrathin sections, and the morphology and structure of the samples were observed by G2F20 (200 KV) for the regions or particles of interest.

### Chemical Analyses

Gas samples (0.1 mL) were regularly taken from headspace of incubations with a pressure-lock precision analytical syringe (Baton Rouge, LA, United States). The concentrations of CH_4_ and CO_2_ were analyzed using gas chromatographs GC-7890 (Agilent Technologies, United States) equipped with a thermal conductivity detector (Fu et al., 2018). Liquid samples (0.5 mL) were taken with sterile syringes and centrifuged for 15 min at 17,949 ×*g* at 4°C. The supernatant was collected, passed through 0.22-μm-pore-size filters, and analyzed for the concentrations of acetate and butyrate with an HPLC-1200 using a Zorbax SB-AQ C_18_ column (Agilent Technologies, United States) (Fu et al., 2018). Redox potential (ORP) were measured with an Unisense redox microelectrode RD 100 (Unisense, Denmark).

## Results

### NanoFe_3_O_4_ Specifically Promoted Acetotrophic Methanogenesis in Enrichment Cultures

**FIGURE 1:**
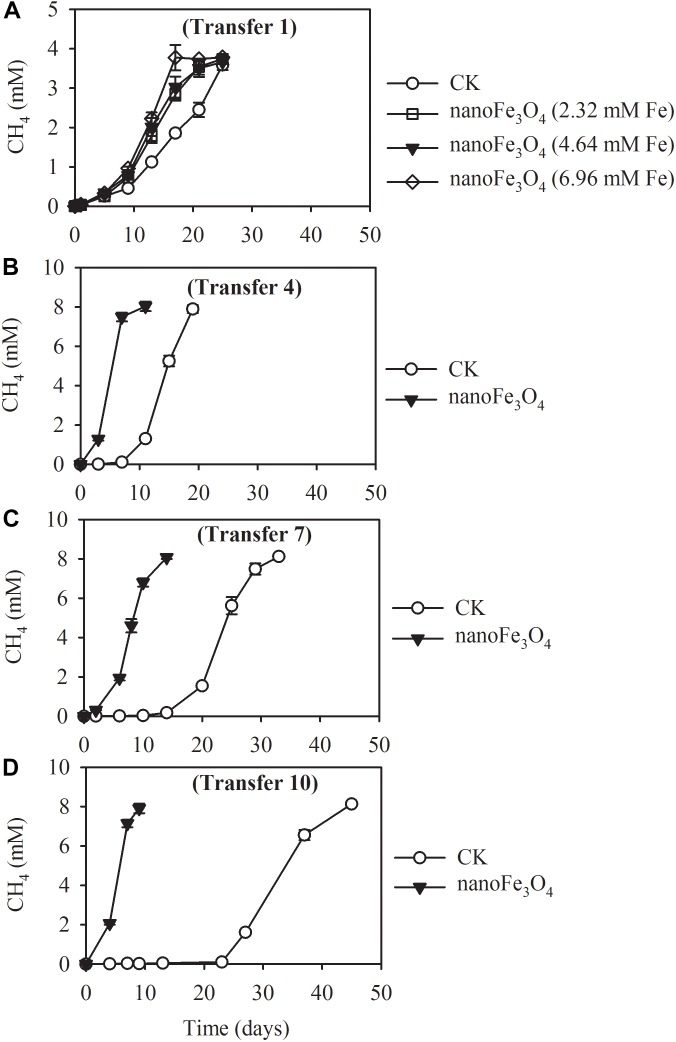
Methane production from acetate in the 1st **(A)**, 4th **(B)**, 7th **(C)**, and 10th **(D)** transfer of wetland enrichment in the presence (nanoFe_3_O_4_) and absence (CK) of nanoFe_3_O_4_. The 1st transfer were inoculated from the pre-incubated soil slurry and the later transfers were inoculated always from the previous nanoFe_3_O_4_-amended transfer. The concentration of CH_4_ produced was expressed as micromole per liter (mM) of the incubation medium. The error bars indicate the standard deviations of three replications.

The production of CH_4_ occurred without lag in the first transfer indicating the ready activity of acetate oxidation in this wetland sediment (Figure 1A). Addition of 5 mM acetate yielded about 3.78 ± 0.13 mM CH_4_ (normalized to liquid volume) in the first transfer (Figure 1A). When the concentration of acetate was increased to 10 mM, about 8.13 ± 0.12 mM of CH_4_ was obtained in the later transfers (Figure 1B–D). These results indicated that CH_4_ accumulated in the headspace corresponded to 80% of theoretical stoichiometric prediction of complete conversion of acetate to CH_4_ and CO_2_. Addition of nanoFe_3_O_4_ did not influence this conversion efficiency. However, nanoFe_3_O_4_ significantly accelerated the CH_4_ production rate, with shorter lags and greater maximal rates compared with the control. For the 4th transfer, CH_4_ production displayed a long lag in the control while it took less than a week before the onset of rapid production in the presence of nanoFe_3_O_4_ (Figure 1B–D).

**FIGURE 2:**
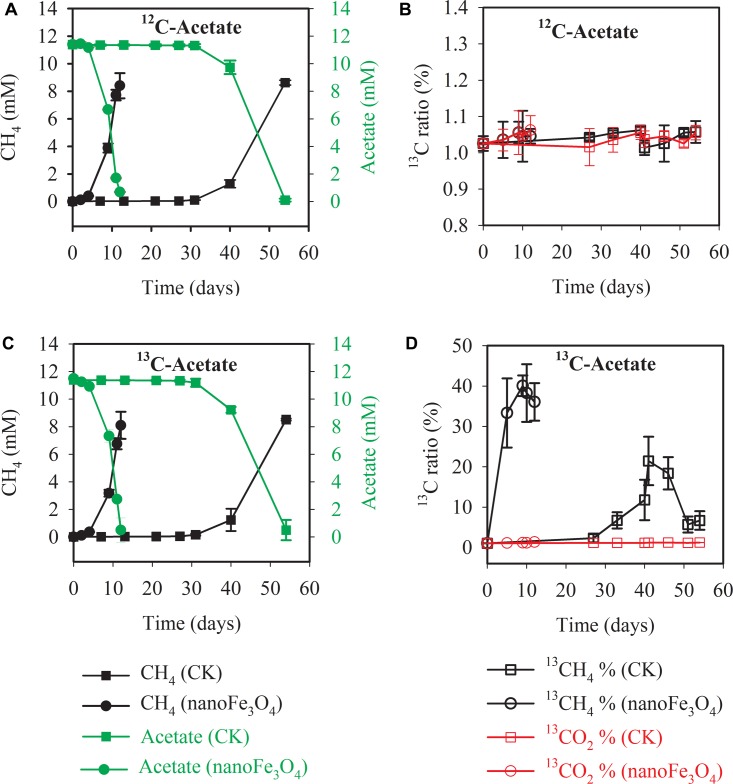
Methane production and acetate consumption in the ^13^C isotope experiments with the 14th transfer enrichment. The sodium acetate-2-^13^C (^13^CH_3_COONa) was used for the labeling treatment. The total concentrations of acetate and CH_4_ were colored in green and black, respectively **(A,C)**. The ^13^CH_4_ percentages in total CH_4_ and ^13^CO_2_ percentages in total CO_2_ were colored in black and red, respectively **(B,D)**. The error bars indicate the standard deviations of three replicates.

Isotopic and chemical analysis was conducted during the labeling experiment at the 14th transfer. Incubations with or without labeling showed identical patterns of acetate consumption and CH_4_ production (Figure 2A without and Figure 2C with isotopic labeling). In consistence with the early transfer incubation experiments, the addition of 10 mM acetate produced about 8.1–8.9 mM CH_4_ (Figure 2A,C). When the sodium acetate-2-^13^C (99 atom%; Sigma-Aldrich) was added as the substrate, the carbon isotopic ratios (δ^13^C values) of CH_4_ increased rapidly in the presence of nanoFe_3_O_4_, peaked at 40 ± 2.5% on the 9th day of culture (Figure 2D). However, the δ^13^C values of CO_2_ for the control (1.3 ± 0.04%) only slightly increased (Figure 2B). These results indicated that CH_4_ was mainly produced from direct acetate cleavage. In the direct pathway, acetotrophic methanogens like *Methanosarcina* and *Methanosaeta* convert acetate into CH_4_ and CO_2_ (^13^CH_3_COOH →^13^CH_4_ + CO_2_). However, in the syntrophic pathway, acetate is oxidized to H_2_ and CO_2_ (^13^CH_3_COOH + 2H_2_O → 4H_2_ + ^13^CO_2_ + ^12^CO_2_). H_2_ is consumed by hydrogenotrophic methanogens to generate CH_4_ (4H_2_ + ^12/13^CO_2_ →^12/13^CH_4_ + 2H_2_O). If the system is dominated by the syntrophic pathway, considering that the headspace of the serum bottle was filled with N_2_:CO_2_ (80:20), the carbon isotopic ratios (δ^13^C values) of CO_2_ will increase, and the carbon isotopic ratios (δ^13^C values) of CH_4_ should be even lower than the carbon isotopic ratios (δ^13^C values) of CO_2_.

**FIGURE 3:**
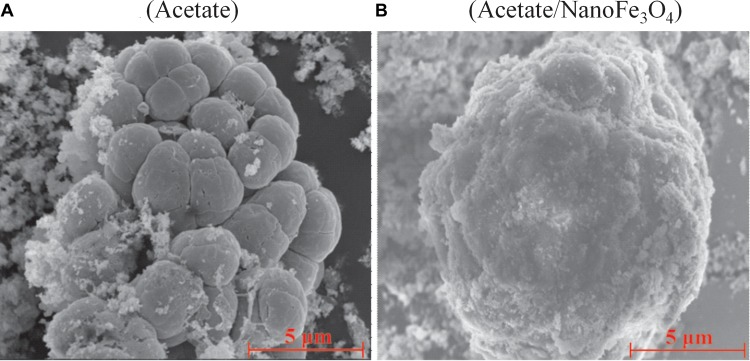
The scanning electron micrography images of the 14th transfer enrichments: **(A)** in the control without nanoFe_3_O_4_ and **(B)** with the amendment of nanoFe_3_O_4_. Scale bars: 5 μm.

Scanning electron micrograph showed the aggregated cells in the control (Figure 3A) at the 14th transfer. With the addition of nanoFe_3_O_4_, the shell-like coat formed on microbial aggregates with nanoFe_3_O_4-_coated surfaces (Figure 3B).

### *Methanosarcina* spp. Are Highly Enriched in Enrichment Cultures Supplemented With NanoFe_3_O_4_

**FIGURE 4:**
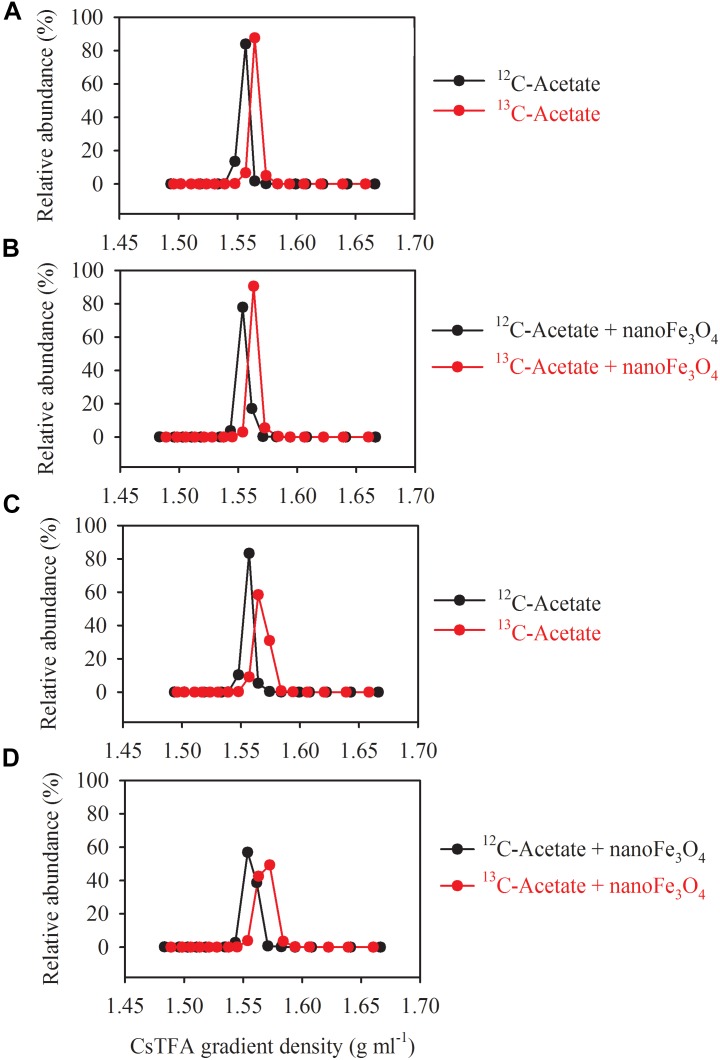
Quantitative profiles of archaeal **(A,B)** and bacterial **(C,D)** 16S rRNA genes across density-gradient fractions in incubations with ^13^C-labeled (red) acetate and the non-labeled control (black), in the presence **(B,D)** or absence **(A,C)** of nanoFe_3_O_4_. The gradient fractions, characterized by their buoyant densities were generated using CsTFA density gradient centrifugation of DNA.

DNA-SIP, T-RFLP and clone sequence analyses were used to determine microbial composition of the enrichment cultures. DNA-SIP was performed by applying ^13^C-labeled acetate (^13^CH_3_COONa). Almost identical pattern was observed in the distribution of the density-resolved DNA fragments along the buoyant density gradient for the control (Figure 4A,C) and the nanoFe_3_O_4_ treatment (Figure 4B,D). The distribution of the archaeal (Figure 4A,B) and bacterial (Figure 4C,D) DNA shifted to the heavier fractions in the labeled samples compared with the non-labeled control (Figure 4). These results indicated that both the archaeal and bacterial populations assimilated ^13^C-labeled acetate.

**FIGURE 5:**
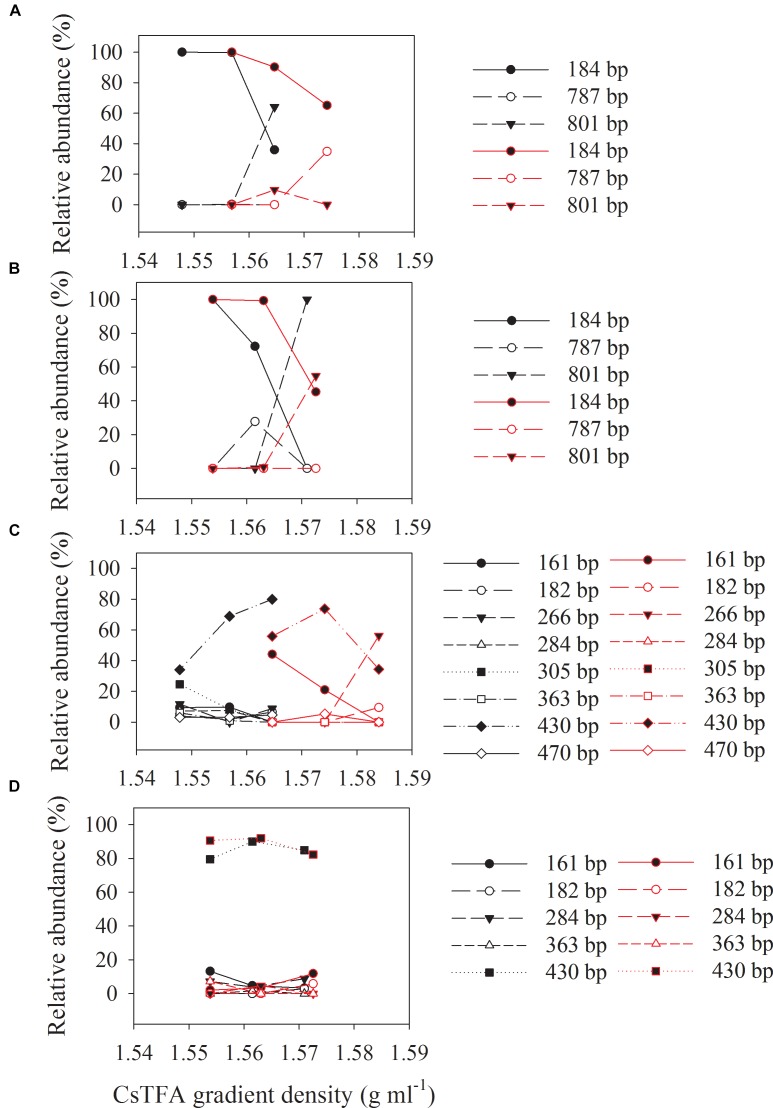
The relative abundances of archaeal **(A,B)** and bacterial **(C,D)** T-RFs in the density-resolved DNA fractions in incubations with ^13^C-labeled (red) acetate and the non-labeled control (black), in the presence **(B,D)** or absence **(A,C)** of nanoFe_3_O_4_. The gradient fractions, characterized by their buoyant densities were generated using CsTFA density gradient centrifugation of DNA.

**FIGURE 6:**
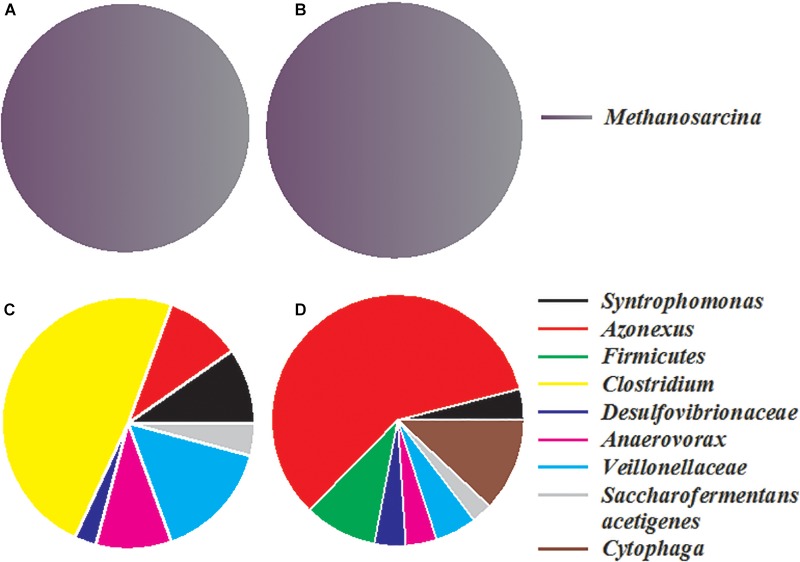
The relative abundances of archaeal **(A,B)** and bacterial **(C,D)** 16S rRNA gene clones generated from the 14th transfer enrichments in the presence **(B,D)** or absence **(A,C)** of nanoFe_3_O_4_.

Three major T-RFs were detected in the archaeal T-RFLP fingerprints (Figure 5A,B) of the density-resolved DNA. The 184 bp T-RF was predominant in all T-RFLP profiles, and its relative abundance was reduced only in heavier layers. Analysis of the clone sequences indicated that these T-RFs belonged to *Methanosarcina* (Figure 6A,B). The T-RFs detected in the bacterial T-RFLP fingerprints include: 167 bp, representing *Anaerovorax*; 182 bp, representing *Syntrophomonas*; 284 bp, representing *Veillonellaceae*; 305 and 470 bp, both representing *Clostridium*; 363 bp, representing *Cytophaga*; 430 bp, representing *Azonexus*. The 430 bp was predominant in all experimental treatments (Figure 5C,D). For the control without nanoFe_3_O_4_, 430 bp showed a trend of increasing first and then decreasing with the DNA buoyant density (Figure 5C); the 305 and 167 bp was secondary dominated in the non-labeling and labeling treatments, respectively. For the nanoFe_3_O_4_ treatments, the 430 bp T-RF was predominant across density gradients; other T-RFs either declined or did not change with the DNA buoyant density (Figure 5D).

Two bacterial and two archaeal clone libraries were constructed, with one each for the control and nanoFe_3_O_4_ treatment, respectively. All the archaeal clone sequences from both the nanoFe_3_O_4_ treatment and the control were affiliated to the *Methanosarcinales* order, with *Methanosarcina barkri* as the closest pure culture relative (Figure 6A,B). Clone sequences indicated that the bacterial communities in the enrichments consisted mainly of *Syntrophomonas, Veillonellaceae, Anaerovorax, Clostridium, Cytophaga, Azonexus, Desulfovibrionaceae*, and *Saccharofermentans acetigenes* (Figure 6C,D). The sequences affiliated to *Azonexus* accounted for approximately 59% of total sequences in the nanoFe_3_O_4_ library (Figure 6D). By comparison, *Clostridium* accounted for 49%, followed by *Veillonellaceae* (15%), *Anaerovorax* (10%) and *Azonexus* (10%) in the control library (Figure 6C).

### NanoFe_3_O_4_ Promote Acetotrophic Methanogenesis by Pure Cultures of *Methanosarcina barkeri*

**FIGURE 7:**
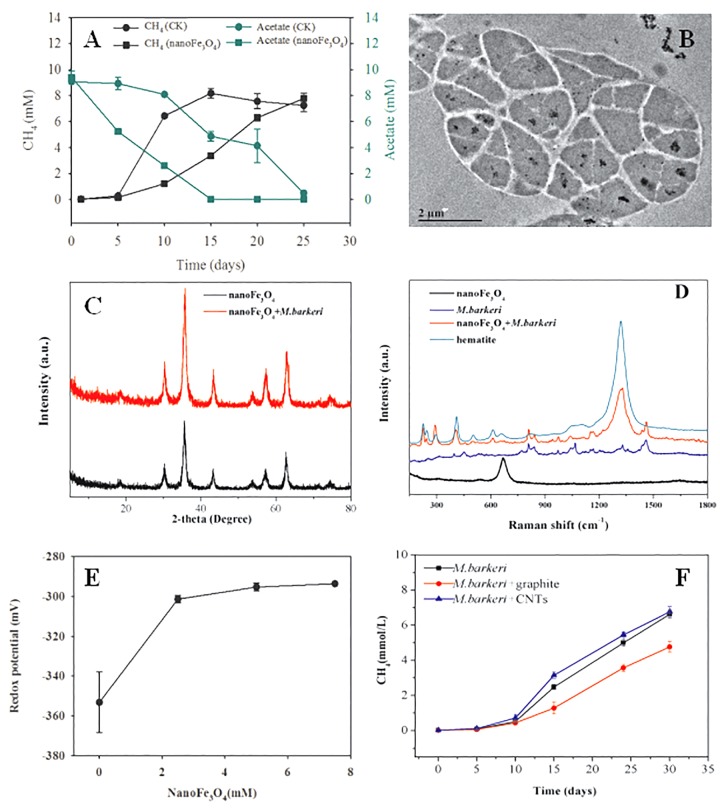
Effect of nanoFe_3_O_4_ on CH_4_ production by *M. barkeri.* **(A)** The total concentrations of CH_4_ (black) and acetate (green) with nanoFe_3_O_4_ (circle) or without nanoFe_3_O_4_ (square). **(B)** the transmission electron microscope (TEM) image of ultrathin sections of *M. barkeri* with the amendment of nanoFe_3_O_4_. **(C)** The X ray diffraction spectrum of nanoFe_3_O_4_ before the incubation without *M. barkeri* (black) and after the incubation with *M. barkeri* (red). **(D)** The Raman spectrum of *M. barkeri* (blue), nanoFe_3_O_4_ before the incubation without *M. barkeri* (black) and after the incubation with *M. barkeri* (red). **(E)** Oxidation-reduction potential (ORP) changes in medium with different concentrations of nanoFe_3_O_4_. **(F)** Methane production from acetate by *M. barkeri*. Three treatments were applied with the additions of: graphite nanoparticles (*M. barkeri* + Graphite), carbon nanotubes (*M. barkeri* + CNTs), and the control without nanomaterials (*M. barkeri*). The error bars indicate the standard deviations of three replicates.

Pure culture systems of *M. barkeri* were constructed with acetate as the sole substrate. Various tests were carried out to investigate the effect of nanoFe_3_O_4_ on the methangenesis of pure culture strains. NanoFe_3_O_4_ significantly promoted the production of methane and the consumption of acetate by *M. barkeri* (Figure 7A). The maximum methane production rate (*V_max_*) was 6.15 ± 0.34 mmol/L⋅d in the presence of nanoFe_3_O_4_, which was about two times that of the control (2.91 ± 0.21 mmol/L⋅d). This rapid methanogenesis process occurred 10 days earlier than the control. However, the addition of nano-graphite (particle diameter: 35 nm) did not show any promoting effect. On the other hand, the addition of CNTs (outside diameter: 10–20 nm, length: 10–30 μm) retained a stimulatory effect that was less significant compared with nanoFe_3_O_4_ (Figure 7F).

### Redox Activity Rather Than Electrical Conductivity of NanoFe_3_O_4_ Leads to Enhanced Methanogenesis by *M. barkeri*

In order to clarify the action sites of nanoFe_3_O_4_ on *M. barkeri*, we performed ultrathin sections of *M. barkeri* and TEM analysis. The results showed that the nanoFe_3_O_4_ existed in extracellular and intercellular spaces, cell surfaces, cell membranes, and the cytoplasms (Figure 7B). XRD was used to determine the possible structural changes of nanoFe_3_O_4_ during the culture process. The XRD pattern data showed no change in the crystal structure of nanoFe_3_O_4_ after culture (Figure 7C). The structure and composition of nanoFe_3_O_4_ were further analyzed with Raman spectroscopy on a microscopic scale. The Raman spectrum detected the production of hematite (compared to a Raman spectrum of hematite reported in the RRUFF data base^1^) after the incubation with *M. barkeri* (Figure 7D).

**FIGURE 8:**
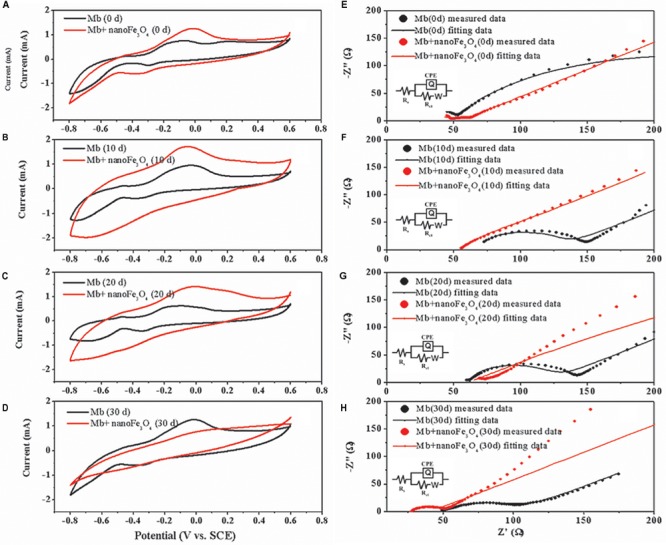
Cyclic voltammograms of *M. barkeri* culture system with (red) or without nanoFe_3_O_4_ (black) at 0 **(A)**, 10 **(B)**, 20 **(C)**, and 30 **(D)** days. Electrochemical impedance spectra of *M. barkeri* culture system with (red) or without nanoFe_3_O_4_ (black) at 0 **(E)**, 10 **(F)**, 20 **(G)**, and 30 **(H)** days.

After the addition of nanoFe_3_O_4_, the oxidation-reduction potential (ORP) of the culture system was increased from -350 to -300 mV, but did not continue to increase as the concentration of nanoFe_3_O_4_ increased (Figure 7E). We performed a cyclic voltammogram (CV) scan of all pure culture methanogenic systems to analyze the redox activity changes of the systems. CV can reflect the redox activity and capacitance in the electrochemical reactor. The larger the capacitance, the more charge is stored in the system. As shown in Figure 8A–D, the capacitance of the bioreactors with nanoFe_3_O_4_ was higher than that of the control reactors, suggesting an enhanced redox activity. The capacitance of the bioreactors with nanoFe_3_O_4_ increased to the maximum on day 10 (Figure 8B) and then gradually decreased to the minimum on day 30 (Figure 8D), which was consistent with the methanogenic activity. By contrast, the capacitance of the control bioreactors without nanoFe_3_O_4_ kept relatively stable during the 30-days cultivation. Moreover, a pair of oxidation and reduction peaks with a mid-point potential of approximately -200 mV vs. SHE appeared in the CV scans for both treatments. However, the peaks disappeared for the bioreactors with nanoFe_3_O_4_ on day 30. This indicated the redox transformation of certain redox-active species in the bioreactors with nanoFe_3_O_4_. EIS was used to evaluate the electron transfer resistance of the reactors, which was crucial for the charge transfer efficiency. As shown in Figure 8E–H, the addition of nanoFe_3_O_4_ significantly decreased the electron transfer resistance (R_ct_ = 37.7 Ω) compared with the control reactors (R_ct_ = 353.5 Ω) at day 0. Although the electron transfer resistances of the control reactors decreased to 68.9 Ω after 30-days cultivation it was still higher than that for the nanoFe_3_O_4_ treatment (27.6 Ω). The decreasing R_ct_ over time might be due to the adsorption of *M. barkeri* on the electrode surfaces.

## Discussion

### Redox Behavior of Magnetite in NanoFe_3_O_4_/*M. barkeri* Interface

**FIGURE 9:**
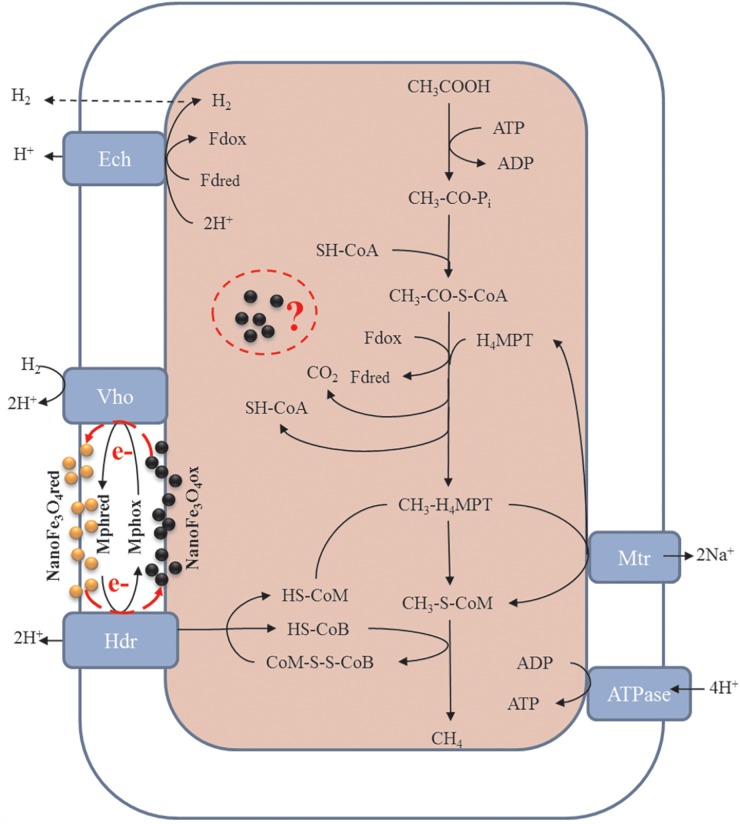
Proposed hypothesis of nanoFe_3_O_4_ as solid electron shuttles for acetotrophic methanogenesis by *M. barkeri.* Black and yellow spheres indicate oxidized and reduced nanoFe_3_O_4_, respectively. Adapted from Ferry (2010).

Previous literatures have reported that conductive materials such as nanoFe_3_O_4_, graphite, CNTs, activated carbon, etc., can promote the methane production via mediating DIET. However, few studies have focused on the effects of these materials on methanogens themselves. This work evaluated the effects of nanoFe_3_O_4_ in enrichment cultures initiated with wetland soils with acetate as a substrate. Compared with the control, the addition of nanoFe_3_O_4_ consistently shortened the lag period and enhanced the maximum rate of CH_4_ production. Highly enriched methanogens were obtained through continuous transfers in the presence of nanoFe_3_O_4_. Mass balance and isotopic labeling indicated that the conversion of acetate conformed to the formula: CH_3_COOH → CH_4_ + CO_2_. Molecular analyses revealed that *Methanosarcina* closely related to a *M. bakeri* strain were left as the only methanogen in the enrichment. These results suggested that nanoFe_3_O_4_ played an important role in the acetotrophic methanogenesis (Figure 9). Such a promoting effect of nanoFe_3_O_4_ was further confirmed in pure culture of *M. bakeri.*

Different mechanisms may be involved in the promoting effect of nanoFe_3_O_4_/*M. bakeri* system. Firstly, nanoFe_3_O_4_ has a relatively low redox potential (-314 mV) (Straub et al., 2001). It has been argued that the stimulatory effect by nanomaterials like CNTs on syntrophic coculture and pure culture of methanogens is caused by the decrease in redox potential (Salvador et al., 2017). Similar effect may be postulated for nanoFe_3_O_4_. However, the experimental results showed that the ORP of the system increased about 50 mV after the addition of nanoFe_3_O_4_ (Figure 7E). Therefore, this inference was untenable here.

Our previous research suggested that the electrical conductivity of nanomaterials played the key role in promoting the syntrophic oxidation of butyrate (Li et al., 2015; Fu et al., 2018). However, the results of this study showed that graphite and CNTs with higher conductivity did not have the similar promoting effect like nanoFe_3_O_4_ (Figure 7F). Apart from the common property in electric conductivity, nanoFe_3_O_4_, CNTs and graphite are chemically and physically different. It was probable that a certain special property of nanoFe_3_O_4_ rather than the conductivity played a decisive role.

The system’s CV scan results showed that the redox activity was highly consistent with the methanogenic activity. The addition of nanoFe_3_O_4_ significantly increased the redox activity of the system (Figure 8A–D). Since soluble electron shuttle molecule was not present in our system, the CV measurement could reflect the activities of the enzymes that catalyzed the electron transport in the membrane. In other words, the higher catalytic currents could be an indicator of a better redox enzyme activity. As shown in Figure 8A–D, the catalytic currents for the nanoFe_3_O_4_ treatment increased in the first 10 days and then gradually decreased to the background at day 30. This means that the redox enzyme activities in the membrane were the highest in the middle period of the experiments. The detection of hematite by Raman further indicated that nanoFe_3_O_4_ did undergo a redox transformation on the microcosmic scale (Figure 7D). Based on this, we can infer that nanoFe_3_O_4_ participate in the electron transfer on the membrane of *M. bakeri*.

### NanoFe_3_O_4_ as Solid-State Electron Shuttles for Enhanced Acetotrophic Methanogenesis

Acetate is the worst substrate for methanogenesis with a standard free energy change of only -36 kJ mol^-1^. It can be only used by cytochrome-containing methanogens such as *Methanosarcina acetivorans, Methanosarcina mazei, and Methanosarcina barkeri* (Thauer et al., 2008; Welte and Deppenmeier, 2014). To conserve the little energy available as much as possible, acetotrophic methanogens have evolved a sophisticated metabolic pathway (Figure 9). In the pathway, CH_3_COOH reacts with sulfhydryl-containing coenzyme A (SH-CoA) to form CH_3_-CO-S-CoA. This is an endergonic reaction. Then, the carboxyl group combines with the oxidized ferredoxin (Fd_ox)_ to form a reduced ferredoxin (Fd_red_), and releases CO_2_ and regenerates SH-CoA. The methyl moiety combines with tetrahydromethanopterin (H_4_MPT) to form CH_3_-H4MPT, which reacts with sulfhydryl-containing coenzyme M (HS-CoM) to form CH_3_-S-MPT and H_4_MPT. Then CH_3_-S-MPT reacts with sulfhydryl-containing coenzyme B (HS-CoB) to form CH_4_ and heterodisulfide of CoM and CoB (CoM-S-S-CoB) (Costa and Leigh, 2014; Welte and Deppenmeier, 2014). In *Methanosarcina* species, Fd_red_ is used to produce H_2_ by ferredoxin-dependent hydrogenase (Ech). The H_2_ diffuses across the cell membrane and is oxidized by methanophenazine-dependent hydrogenase (Vht) to reduce a membrane-bound methanophenazine (Mph). The reduced Mph delivers electrons to the membrane-bound heterodisulfide reductase (HdrDE) to regenerate free CoM and CoB from the CoM-S-S-CoB (Kulkarni et al., 2018; Lovley, 2018; Mand et al., 2018).

Mph is a unique membrane electron carrier found in *Methanosarcina* species (Beifuss et al., 2000). The Mph-catalyzed step is rate limiting in the central metabolism of acetate. Mph has a lower redox potential (-150 mV). The content of Mph in *M. acetivorans* were threefold higher than that in *M. barkeri* (Duszenko and Buan, 2017). This suggests the cell membrane of *M. acetivorans* is electrically quantized as if it were a single conductive metal sheet and near optimal for electron transport (Duszenko and Buan, 2017). Therefore, in theory, by adding an electron carrier with a function similar to Mph to the medium, the acetotrophic methanogenic rate of *M. barkeri* can be increased. Beckmann et al. have demonstrated that soluble neutral red (-375 mV) delivers reducing equivalents directly to the membrane bound HdrED of *Methanosarcina* species, and thus increases the rates of proton translocation and regeneration of the methanogenic cofactors CoM-SH and CoB-SH (Beckmann et al., 2016). This was the first report that an artificial electron shuttle can mimic the membrane integrated electron shuttle Mph.

Although nanoFe_3_O_4_ (-314 mV) existed in solid state, the results of ultrathin sections proved that nanoFe_3_O_4_ passed through the cell membrane of *M. barkeri*, so nanoFe_3_O_4_ could function as electron shuttles like soluble neutral red. It was worth mentioning that nanoFe_3_O_4_ did not show any effect on CH_4_ production by two hydrogenotrophic methanogens *Methanococcus maripaludis* and *Methanocella conradii* (Fu et al., 2018). The most essential difference between them and *M. barkeri* was the lack of electron transport chain on the membrane (Thauer et al., 2008; Costa and Leigh, 2014; Welte and Deppenmeier, 2014). Based on the characteristics and performance of nanoFe_3_O_4_, we propose the hypothesis that nanoFe_3_O_4_ may act as solid-state electron shuttles that performs a similar function like Mph. Here we also emphasize that this may not be the only mechanism by which nanoFe_3_O_4_ stimulates methane production. Other unknown biochemical activities may influence methanogenesis. Our experimental evidence has clarified that a large amount of nanoFe_3_O_4_ passes through the cell membrane and enters the cytoplasm. What changes may occur in this part of the nanoFe_3_O_4_, what reactions they may participate in, and what kind of effects on the organism, we don’t know anything about these issues and deserve further study.

## Data Availability

The datasets generated for this study can be found in GenBank, MK061905–MK062157.

## Author Contributions

SZ and YL conceived the research. LF performed the enrichment cultivation, isotope labeling, and molecular analysis. TZ and JW performed the pure culture test. LpY performed the electrochemical analysis. LxY performed the Raman analysis. LF wrote the manuscript. LF and LpY edited the manuscript. All authors reviewed and approved the manuscript.

## Conflict of Interest Statement

The authors declare that the research was conducted in the absence of any commercial or financial relationships that could be construed as a potential conflict of interest.

## Footnotes

1 http://rruff.info/hematite/display=default/R040024

## References

[B1] Beckmann S. Welte C. Li X. Oo Y. M. Kroeninger L. Heo Y. (2016). Novel phenazine crystals enable direct electron transfer to methanogens in anaerobic digestion by redox potential modulation. *Energy Environ. Sci.* 9 644–655. 10.1039/C5EE03085D

[B2] Beifuss U. Tietze M. Bäumer S. Deppenmeier U. (2000). Methanophenazine: structure, total synthesis, and function of a new cofactor from methanogenic *archaea*. *Angew. Chem. Int. Ed.* 39 2470–2472. 10.1002/1521-3773(20000717)39:14<2470::AID-ANIE2470>3.0.CO;2-R 10941105

[B3] Costa K. C. Leigh J. A. (2014). Metabolic versatility in methanogens. *Curr. Opin. Biotechnol.* 29 70–75. 10.1016/j.copbio.2014.02.012 24662145

[B4] Duszenko N. Buan N. R. (2017). Physiological evidence for isopotential tunneling in the electron transport chain of methane-producing *Archaea*. *Appl. Environ. Microbiol.* 83 AEM.00950–AEM.01017. 10.1128/AEM.00950-17 28710268 PMC5583484

[B5] Ferry J. G. (2010). CO in methanogenesis. *Ann. Microbiol.* 60 1–12. 10.1007/s13213-009-0008-5

[B6] Fleet M. (1981). The structure of magnetite. *Acta Crystallograp. Sec. B* 37 917–920. 10.1107/S0567740881004597

[B7] Fu L. Song T. Lu Y. (2015). Snapshot of methanogen sensitivity to temperature in zoige wetland from tibetan plateau. *Front. Microbiol.* 6:131. 10.3389/fmicb.2015.00131 25745422 PMC4333864

[B8] Fu L. Song T. Zhang W. Zhang J. Lu Y. (2018). Stimulatory effect of magnetite nanoparticles on a highly enriched butyrate-oxidizing consortium. *Front. Microbiol.* 9:1480. 10.3389/fmicb.2018.01480 30026737 PMC6041394

[B9] Gan Y. Qiu Q. Liu P. Rui J. Lu Y. (2012). Syntrophic oxidation of propionate in rice field soil at 15 and 30 C under methanogenic conditions. *Appl. Environ. Microbiol.* 78 4923–4932. 10.1128/AEM.00688-12 22582054 PMC3416378

[B10] Hao L. P. Lü F. He P. J. Li L. Shao L. M. (2010). Predominant contribution of syntrophic acetate oxidation to thermophilic methane formation at high acetate concentrations. *Environ. Sci. Technol.* 45 508–513. 10.1021/es102228v 21162559

[B11] Hudson R. Feng Y. Varma R. S. Moores A. (2014). Bare magnetic nanoparticles: sustainable synthesis and applications in catalytic organic transformations. *Green Chem.* 16 4493–4505. 10.1039/C4GC00418C

[B12] Jing Y. Wan J. Angelidaki I. Zhang S. Luo G. (2017). iTRAQ quantitative proteomic analysis reveals the pathways for methanation of propionate facilitated by magnetite. *Water Res.* 108 212–221. 10.1016/j.watres.2016.10.077 27817893

[B13] Kang Y. S. Risbud S. Rabolt J. F. Stroeve P. (1996). Synthesis and characterization of nanometer-size Fe3O4 and γ-Fe2O3 particles. *Chem. Mater.* 8 2209–2211. 10.1021/cm960157j 16455092

[B14] Kato S. Hashimoto K. Watanabe K. (2012). Methanogenesis facilitated by electric syntrophy via (semi)conductive iron-oxide minerals. *Environ. Microbiol.* 14 1646–1654. 10.1111/j.1462-2920.2011.02611.x 22004041

[B15] Kulkarni G. Mand T. D. Metcalf W. W. (2018). Energy conservation via hydrogen cycling in the methanogenic *Archaeon Methanosarcina barkeri*. *Mbio* 9 e01256–e1318. 10.1128/mBio.01256-18 29970471 PMC6030560

[B16] Kumar S. Stecher G. Tamura K. (2016). MEGA7: Molecular evolutionary genetics analysis version 7.0 for bigger datasets. *Mol. Biol. Evol.* 33 1870–1874. 10.1093/molbev/msw054 27004904 PMC8210823

[B17] Li H. Chang J. Liu P. Fu L. Ding D. Lu Y. (2015). Direct interspecies electron transfer accelerates syntrophic oxidation of butyrate in paddy soil enrichments. *Environ. Microbiol.* 17 1533–1547. 10.1111/1462-2920.12576 25059331

[B18] Liu P. Qiu Q. Lu Y. (2011). Syntrophomonadaceae-affiliated species as active butyrate-utilizing syntrophs in paddy field soil. *Appl. Environ. Microbiol.* 77 3884–3887. 10.1128/AEM.00190-11 21460111 PMC3127591

[B19] Lovley D. R. (2017). Syntrophy goes electric: direct interspecies electron transfer. *Ann. Rev. Microbiol.* 71 643–664. 10.1146/annurev-micro-030117-020420 28697668

[B20] Lovley D. R. (2018). The hydrogen economy of *Methanosarcina barkeri*: life in the fast lane. *J. Bacteriol.* 200 e00445–e00518. 10.1128/JB.00445-18 30082458 PMC6153660

[B21] Lü Z. Lu Y. (2012). *Methanocella conradii* sp nov., a thermophilic, obligate hydrogenotrophic methanogen, isolated from chinese rice field soil. *PLoS One* 7:e35279. 10.1371/journal.pone.0035279 22530002 PMC3328440

[B22] Ma K. Conrad R. Lu Y. (2012). Responses of methanogen mcrA genes and their transcripts to an alternate dry/wet cycle of paddy field soil. *Appl. Environ. Microbiol.* 78 445–454. 10.1128/AEM.06934-11 22101043 PMC3255761

[B23] Mand T. D. Kulkarni G. Metcalf W. W. (2018). Genetic, biochemical, and molecular characterization of *Methanosarcina barkeri* mutants lacking three distinct classes of hydrogenase. *J. Bacteriol.* 200:16. 10.1128/JB.00342-18 30012731 PMC6153667

[B24] Mohammed L. Gomaa H. G. Ragab D. Zhu J. (2017). Magnetic nanoparticles for environmental and biomedical applications: a review. *Particuology* 30 1–14. 10.1016/j.partic.2016.06.001

[B25] Rotaru A.-E. Shrestha P. M. Liu F. Markovaite B. Chen S. Nevin K. P. (2014). Direct interspecies electron transfer between *Geobacter metallireducens* and *Methanosarcina barkeri*. *Appl. Environ. Microbiol.* 80 4599–4605. 10.1128/AEM.00895-14 24837373 PMC4148795

[B26] Rui J. Qiu Q. Lu Y. (2011). Syntrophic acetate oxidation under thermophilic methanogenic condition in chinese paddy field soil. *Fems Microbiol. Ecol.* 77 264–273. 10.1111/j.1574-6941.2011.01104.x 21470253

[B27] Salvador A. F. Martins G. Melle-Franco M. Serpa R. Stams A. J. M. Cavaleiro A. J. (2017). Carbon nanotubes accelerate methane production in pure cultures of methanogens and in a syntrophic coculture. *Environ. Microbiol.* 19 2727–2739. 10.1111/1462-2920.13774 28447396

[B28] Schnürer A. Zellner G. Svensson B. H. (1999). Mesophilic syntrophic acetate oxidation during methane formation in biogas reactors. *FEMS Microbiol. Ecol.* 29 249–261. 10.1016/S0168-6496(99)00016-1

[B29] Straub K. L. Benz M. Schink B. (2001). Iron metabolism in anoxic environments at near neutral pH. *FEMS Microbiol. Ecol.* 34 181–186. 10.1111/j.1574-6941.2001.tb00768.x 11137597

[B30] Su C. (2017). Environmental implications and applications of engineered nanoscale magnetite and its hybrid nanocomposites: a review of recent literature. *J. Hazard. Mater.* 322 48–84. 10.1016/j.jhazmat.2016.06.060 27477792 PMC7306924

[B31] Tang J. Zhuang L. Ma J. Tang Z. Yu Z. Zhou S. (2016). Secondary mineralization of ferrihydrite affect microbial methanogenesis in geobacter/methanosarcina co-cultures. *Appl. Environ. Microbiol.* 82 AEM.01517–1616. 10.1128/AEM.01517-16 27451453 PMC5038019

[B32] Thauer R. K. Kaster A.-K. Seedorf H. Buckel W. Hedderich R. (2008). Methanogenic archaea: ecologically relevant differences in energy conservation. *Nat. Rev. Microbiol.* 6 579–591. 10.1038/nrmicro1931 18587410

[B33] Viggi C. C. Rossetti S. Fazi S. Paiano P. Majone M. Aulenta F. (2014). Magnetite particles triggering a faster and more robust syntrophic pathway of methanogenic propionate degradation. *Environ. Sci. Technol.* 48 7536–7543. 10.1021/es5016789 24901501

[B34] Welte C. Deppenmeier U. (2014). Bioenergetics and anaerobic respiratory chains of aceticlastic methanogens. *Biochim. Et Biophys. Acta Bioenerg.* 1837 1130–1147. 10.1016/j.bbabio.2013.12.002 24333786

[B35] Yamada C. Kato S. Ueno Y. Ishii M. Igarashi Y. (2015). Conductive iron oxides accelerate thermophilic methanogenesis from acetate and propionate. *J. Biosci. Bioeng.* 119 678–682. 10.1016/j.jbiosc.2014.11.001 25488041

[B36] Yang Z. Xu X. Guo R. Fan X. Zhao X. (2015). Accelerated methanogenesis from effluents of hydrogen-producing stage in anaerobic digestion by mixed cultures enriched with acetate and nano-sized magnetite particles. *Biores. Technol.* 190 132–139. 10.1016/j.biortech.2015.04.057 25935393

[B37] Zhang J. Lu Y. (2016). Conductive Fe3O4 nanoparticles accelerate syntrophic methane production from butyrate oxidation in two different lake sediments. *Front. Microbiol.* 7:1316. 10.3389/fmicb.2016.01316 27597850 PMC4992681

[B38] Zhou S. Tang J. Yuan Y. Yang G. Xing B. (2018). TiO2 nanoparticle-induced nanowire formation facilitates extracellular electron transfer. *Environ. Sci. Technol. Lett.* 5 564–570. 10.1021/acs.estlett.8b00275

[B39] Zhuang L. Ma J. Yu Z. Wang Y. Tang J. (2018). Magnetite accelerates syntrophic acetate oxidation in methanogenic systems with high ammonia concentrations. *Microb. Biotechnol.* 11 710–720. 10.1111/1751-7915.13286 29896929 PMC6011935

[B40] Zhuang L. Tang J. Wang Y. Hu M. Zhou S. (2015). Conductive iron oxide minerals accelerate syntrophic cooperation in methanogenic benzoate degradation. *J. Hazard. Mater.* 293 37–45. 10.1016/j.jhazmat.2015.03.039 25827267

